# Mechanisms for estrogen receptor expression in human cancer

**DOI:** 10.1186/s40164-018-0116-7

**Published:** 2018-09-19

**Authors:** Hui Hua, Hongying Zhang, Qingbin Kong, Yangfu Jiang

**Affiliations:** 1 Laboratory of Stem Cell Biology, West China Hospital, Sichuan University, Chengdu, China; 2 Laboratory of Oncogene, West China Hospital, Sichuan University, Chengdu, China

**Keywords:** Cancer, Estrogen receptor, Transcription, Epigenetic modification

## Abstract

Estrogen is a steroid hormone that has critical roles in reproductive development, bone homeostasis, cardiovascular remodeling and brain functions. However, estrogen also promotes mammary, ovarian and endometrial tumorigenesis. Estrogen antagonists and drugs that reduce estrogen biosynthesis have become highly successful therapeutic agents for breast cancer patients. The effects of estrogen are largely mediated by estrogen receptor (ER) α and ERβ, which are members of the nuclear receptor superfamily of transcription factors. The mechanisms underlying the aberrant expression of ER in breast cancer and other types of human tumors are complex, involving considerable alternative splicing of ERα and ERβ, transcription factors, epigenetic and post-transcriptional regulation of ER expression. Elucidation of mechanisms for ER expression may not only help understand cancer progression and evolution, but also shed light on overcoming endocrine therapy resistance. Herein, we review the complex mechanisms for regulating ER expression in human cancer.

## Background

Estrogens are steroidal hormones that function as the primary female sex hormone. There are three major forms of estrogen, namely estrone (E1), estradiol (E2) and estriol (E3). Estradiol (E2) is the predominant estrogen in nonpregnant females, while estrone and estriol are primarily produced during pregnancy and following the onset of menopause [1], respectively. 17-β-estradiol is the primary estrogen from menarche to menopause [2]. All estrogens are produced from androgens through actions of enzymes such as aromatase [3]. Follicle-stimulating hormone and luteinizing hormone stimulate the synthesis of estrogen in the ovaries [4]. However, some estrogens are also produced in smaller amounts by other tissues such as the liver, adrenal glands, and mammary gland [5]. Previous studies suggest that estrogen is associated with mammary tumorigenesis, ovarian and endometrial carcinogenesis [6]. Also, mounting evidence demonstrate that estrogen and its target gne progesterone receptor (PR) play critical roles in regulatiing breast cancer progression and cancer stem cell fate [7, 8]. However, estrogen may have anti-cancer effects in some organs such as the liver and colon, whilst more studies are needed to clarify this argument and better understand the mechanisms [9–12].

The biological effects of estrogen are mostly mediated by its binding and activation of ERα and ERβ, which are members of the nuclear receptor superfamily of transcription factors that are characterized by highly conserved DNA- and ligand-binding domains [3, 13]. The DNA binding domain, which is extremely well conserved between ERα and ERβ (97% homology), contains two functionally distinct zinc finger motifs that are responsible for specific DNA binding, as well as mediating receptor dimerization [3]. The unliganded ER has been shown to be present in a cytosolic complex with hsp90 and associated proteins, with ligand binding allowing dissociation from the hsp90 complex, receptor dimerisation, nuclear localisation and binding to estrogen response elements (ERE, 5′-AGGTCAnnnTGACCT-3′) in promoters of estrogen-regulated genes [14, 15]. Genome-wide chromatin immunoprecipitation studies have confirmed that the majority of ER-binding sites in estrogen responsive genes conform well to this consensus sequence [16]. While ERα and ERβ can bind to most ERE identically, the differences in ERα and ERβ may lead to tethering differential transcription factors and then modulating different target genes [17, 18]. Thus, the activation of ERα or ERβ can produce both unique and overlapping effects.

ERα has also been shown to modulate gene transcription through heterodimerizing with other transcription factors such as activating protein 1 (AP1) and nuclear factor kappa-light-chain-enhancer of activated B cells (NF-*k*B) [19, 20]. There is a large profile of estrogen-responsive genes, including pS2, cathepsin D, c-fos, c-jun, c-myc, TGF-α, retinoic acid receptor α1, efp, progesterone receptor (PR), insulin-like growth factor 1 (IGF1) [21]. Many of these ER-regulated genes, including IGF1, cyclin D1, c-myc, and efp, are important for cell proliferation and survival. C-myc is a bona-fide oncogene that is amplified or overexpressed in a variety of human tumors [22]. Efp is an ubiquitin ligase that promotes proteasomal degradation of 14-3-3 sigma thereby stimulating cellular proliferation [23]. While PR is an estrogen-responsive gene, it may antagonize ERα action to inhibit tumor growth, paticulary through interating with RNA polymerase III and inhibiting tRNA transcription [24].

Notably, a pool of ERα are located in the plasma membrane and cytoplasm [25], where it binds to diverse membrane or cytoplasmic signaling molecules such as the p85 regulatory subunit of class I phosphoinositide 3-kinase, mitogen-activated protein kinase (MAPK) and Src [26, 27]. Activation of these signal transduction pathways by estrogen initiates cell survival and proliferation signals. Additionally, these signaling molecules are able to phosphorylate the ERα and its co-regulators to augment nuclear ERα signaling [28]. The genomic and non-genomic actions of ERα play a crucial role in breast epithelial cell proliferation and survival, as well as mammary tumorigenesis [28]. The purpose of this review is to decipher the complex mechanisms underlying the abberant expression of ERα and ERβ in human cancer.

## Expression of ER in normal tissues

The human ERα and ERβ cDNA were cloned in 1985 and 1986, respectively [29, 30]. In human mammary gland, ERα positive cells are present in ducts and lobules, but not in stromal cells. ERα expression is largely heterogeneous within different areas of breast tissue. Only a small fraction of epithelial cells in ducts and lobules are ERα-positive [31]. The ERα levels in mammary gland are affected by menstrual cycle, with more ERα-positive cells in the follicle stage of the cycle [32]. ERα-positive epithelial cells may promote proliferation of surrounding ERα-negative cells, probably through secretion of paracrine factors [33]. While ERα is expressed in luminal epithelial cells but not in the stroma, ERβ is present in luminal, myoepithelial and stromal cells [7]. Depletion of ERα leads to failure to initiate the pre- and postpubertal stages of mammary gland growth, as well as pregancy-induced maturation [34]. ERβ knockout, however, has little effects on mammary gland development [35]. In addition, ERα expression is also detectable in endometrium and ovary. ERα knockout severely disrupts sexual maturation of the whole reproductive tract [36]. ERβ knockout, however, predominantly disrupts the maturation of ovarian function [37].

ERα and ERβ are both expressed in other cell types, though at lower levels than those found in reproductive tissues. Myeloid and lymphoid progenitor cells, mature lymphocytes, and neutrophils express ERα, ERβ, or both receptors [38]. Although 17-β-estradiol inhibits T and B cell development, it enhances B cell function in ERα-dependent manner, involving both genomic and non-genomic ER signaling in B lymphocytes [39, 40]. Moreover, the brain of both sexes is a major target of estradiol and a site of estrogen synthesis [41, 42]. ERβ is a dominant ER subtype in the adult cerebellum. ERβ expression was detected in Golgi type neurons, Purkinje cells, and basket cells in the adult cerebellum [43]. High levels of ERα expression are also found in the hypothalamus, with particularly elevated expression within the medial preoptic area, as well as the amygdala and ventral medial hypothalamus [44]. Estrogen and its receptors may improve memory and social behaviors, regulate brain lipid metabolism and prevent cortical damage following an ischemic episode [45–47]. In addition, estrogen and ER are important for bone homeostasis, hepatic lipid metabolism and reverse cholesterol transport [48, 49]. While the liver predominatly expresses ERα, the gastointestinal tract and the lung exclusively harbour ERβ [50]. Moreover, both ERα and ERβ are expressed in platelets [51]. An estrogen analogue is able to induce platelets apoptosis and autophagy [52]. Therefore, ER is a vital hormone receptor for human health.

## Expression of ER in human tumors

Based on the ER status, breast tumors can be classified as ER-positive and ER-negative. About 75% of breast cancer cases are ERα positive at diagnosis [53]. Luminal A and basal subtypes are two major subtypes of human breast cancer. ERα is more frequently expressed in luminal A tumors than in basal tumors [54]. ERα-positive cases are not only responsive to endocrine therapies, but also sensitive to CDK4/6 inhibitors [55, 56]. Thus, ER positivity may be associated with a better prognosis [57]. ERα-negative tumors, on the other hand, are more aggressive and metastatic [58]. Importantly, ERα expression in breast cancer is dynamic and reversible. About 50% of patients with ERα-positive primary breast tumors that relapse after adjuvant endocrine therapy have recurrent tumors in which ERα expression is lost [59]. The ERα-negative and human epidermal growth factor receptor 2 (HER2)-positive breast cancer can be treated by HER2-targeting agents [60]. Some ERα-negative breast tumors that are treated with growth factor receptor inhibitors may reexpress ERα and respond to endocrine therapy [61]. Except for ERα, various ERβ isoforms are expressed in breast cancer. Both ERβ1 and ERβ2/cx repress the transcriptional activity of ERα [62].

In general, ERα and ERβ differentially contribute to carcinogenesis and tumor progression with ERα as an oncogene and ERβ as a tumor suppressor. However, some ERβ isoforms, such as ERβ5, may act as oncogene [63]. Studies on the distribution of estrogen receptor (ER) subtypes in ovarian tumors demonstrated that 40–60% of ovarian cancers express ERα, especially in serous tumors and in metastasis [64, 65]. However, less than 20% of patients (ranging from 7 to 18%) respond clinically to anti-estrogen treatment [66]. ERβ expression, however, is significantly higher in normal ovary tissues compared with ovarian carcinoma [67]. Advanced colon cancer is associated with a loss of ERβ, the predominant ER in colon tissue [11]. ERβ specific agonists have anti-cancer effects on colon cancer [12]. In addition, estrogen increases the risk of endometrial carcinoma [64]. It appears that ERα is more frequently expressed in lower grade of endometrial carcinoma [68]. ERα expression in endometrial carcinoma is inversely associated with lymph node metastasis [69].

Previous study demonstrated that both ERα and ERβ were overexpressed in a proportion of hepatitis C virus (HCV)-related hepatocellular carcinoma (HCC) [70]. However, another study shows that the expression of ERα and ERβ were lower in HCC tissues than in normal liver tissues [71]. The expression of ERα was lower in HCC with portal vein tumor thrombus (PVTT) than those without PVTT, suggesting that ERα-positive HCC is less aggressive [72]. In addition, a specific isoform of ERα, ERα-36, is overexpressed in HCC [73]. Estrogen stimulates HCC cells growth through ERα-36 [74]. Therefore, variance in ERα subtypes and isoforms may dictate the response of HCC to estrogen.

## Alternative splicing of ER

**Fig. 1:**
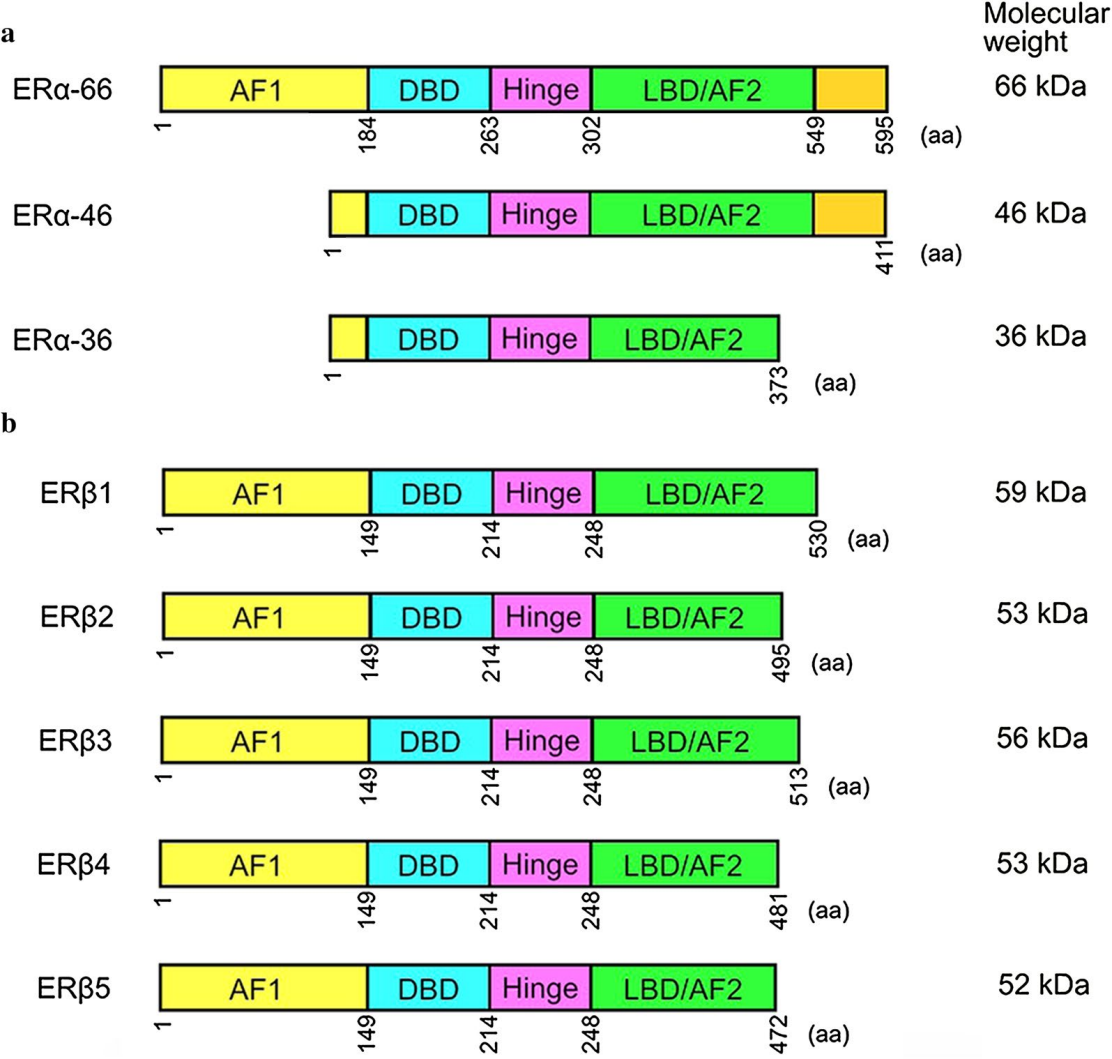
The major ERα and ERβ isoforms. **a** There are three major isoforms of ERα, including ERα-66, ERα-46 and ERα36. ERα-36 differs with ERα-46 in the C-termini. **b** ERβ has five major isoforms, namely ERβ1, ERβ2, ERβ3, ERβ4 and ERβ5. *AF1* activation function 1, *AF2* activation function 2, *DBD* DNA-binding domain, *LBD* ligand-binding domain

Alternatively spliced ERα mRNA has been detected in both normal and cancerous tissues [71, 75]. Variances in ERα transcripts may lead to loss of ligand-dependent transactivation activity, gain of ligand-independent transactivation activity, and differential response to tamoxifen [76]. The most characterized isoform of ERα is a 66-kDa protein encoded by a 6.6-kb mRNA with eight exons [76]. There are six human ERα mRNA isoforms that encode the same 66-kDa protein but differ in their 5′ untranslated region. Moreover, other variant isoforms of ERα mRNA that encode different proteins from the 66-kDa protein can occur in the presence or absence of wild-type ERα transcript (Fig. 1a). The variance in ERα mRNA may be attributed to frame-shift mutations or alternative splicing [77]. A genomic rearrangement in which ERα exons 6 and 7, which encode part of the ligand-binding domain of ERα, are duplicated in an in–frame fashion results in an ERα mRNA that can be translated into a 80 kDa ERα [78]. In addition, a 46-kDa amino-terminal truncated form of ERα, ERα-46, has been identified in endothelial cells and breast cancer cells [75]. ERα-46 is encoded by an ERα transcript that lacks the first exon of the ERα gene [79]. The high monility group A protein 1a (HMGA1a) induces alternative slicing of ERα thereby increasing ERα-46 expresison and reducing tamoxifen sensitivity in breast cancer cells [80]. Mechanistically, HMGA1 traps U1 snRNP at the 5′ splice site of exon 1 in ERα gene thereby inducing alternative splicing [81]. Moreover, some normal or cancer tissues may express the ERα variant that is lack of exon 7 [82]. While the splicing factor HTRA2-β1 is responsible for ERα exon 7 inclusion, heterogeneous nuclear ribonucleoprotein (hnRNP) G induces exon 7 skipping and then promotes the generation of the exon 7-skipping isoform of ERα [83]. In addition, a 36-kDa spliced variant of ERα, ERα-36, has been cloned. ERα-36 is defective of exons 1, 7 and 8, which encode transcriptional activation domains AF1 and AF2 [73]. Both ERα-46 and ERα-36 are located in the plasma membrane, cytosol, and nucleus. ERα-46 and ERα-36 can mediate, at least in part, the membrane-initiated estrogen-dependent activation of mitogenic signaling pathways [27]. ERα-36 also negatively regulates the transactivation activity of ERα-66 and ERβ [84]. Finally, overexpression of the nuclear protein E3-3 (NPE3-3) promotes the generation of another alternatively spliced variant of ERα, ERαV, which contains only exons 1, 2, 7 and 8, and encodes a 37-kDa ERα [85]. Notably, NPE3-3 interacts with multiple splicing factors, including serine/arginine-rich protein (SRp)-30c, SRp40, and splicing factor SC-35 [85].

Variant isoforms of ERβ have also been identified in both normal and cancerous tissues (Fig. 1b). Alternative splicing of exon 8 in the ERβ gene results in five ERβ isoforms (ERβ1, ERβ2, ERβ3, ERβ4 and ERβ5) [86]. The originally cloned ERβ transcript is termed as ERβ1, which is the only isoform that is fully functional. The levels of ERβ1 are low in many tissues, while ERβ2 (also known as ERβcx) is expressed in many tissues and aggressive cancer [87–91]. ERβ4 and ERβ5, however, are predominantly expressed in the testis and placenta, respectively [90]. Both ERβ4 and ERβ5 bind to promoter sequences of DNA but do not bind estrogen. ERβ2, ERβ4 and ERβ5 can heterodimerize with ERα and negatively regulate its transactivation activity [91]. ERβ1 overexpression is associated with better survival in women with breast cancer [92, 93]. Cytoplasmic expression of ERβ2 is associated with poor overall survival in patients with breast cancer and serous ovarian carcinoma [94, 95]. While ERβ1 has tumor suppressive effects on glioblastoma, ERβ5 exhibits oncogenic effects on this type of cancer [62]. Moreover, ERβ5 is associated with poor outcome in HER2-positive and triple-negative breast cancer patients [96]. Taken together, it appears that the ERβ isoforms have different roles in tumorigenesis. Identification of the relative levels of ERβ isoforms may help predict the prognosis in cancer patients. The mechanisms underlying the alternative splicing of ERβ remains largely unknown. The RNA-binding protein Nova1 can bind to the consensus sequences in the ERβ pre-mRNA transcript and then promote exon exclusion of the ERβ2-specific nucleotide sequence, which in turn abolishs ERβ2 mRNA expression but increases ERβ1 mRNA expression [97].

## Transcriptional regulation of ER expression

### Transcription factors that regulate ER expression

**Fig. 2:**
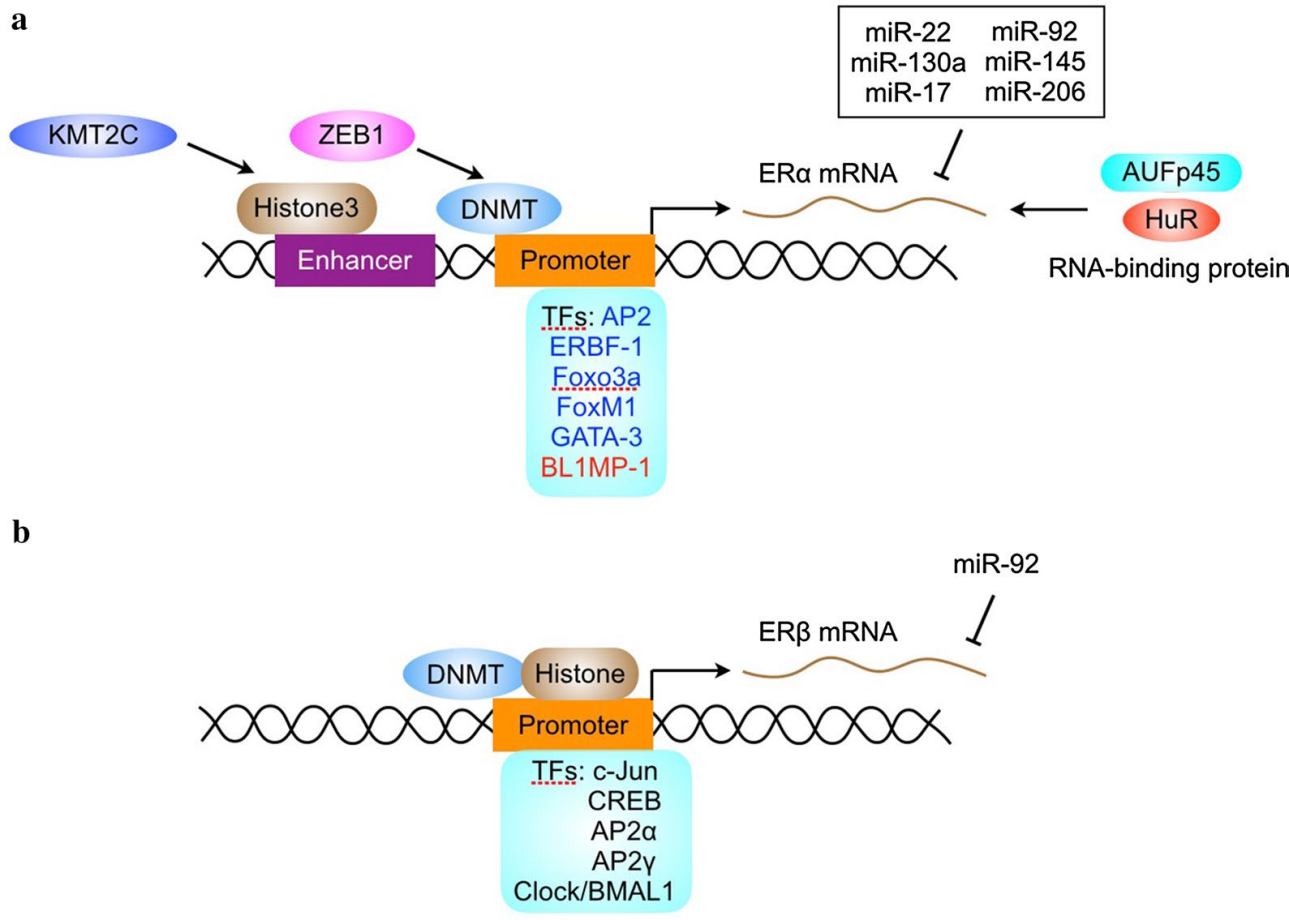
Mechanisms for regulating ERα and ERβ expression. The expression of ERα (**a**) and ERβ (**b**) is regulated by transcription factors (TFs), DNA methylation, histone modification, RNA-binding proteins and miRNA

The human ERα gene spans approximately 300 kb of chromosome 6, including the 140 kb containing the eight protein-coding exons. Since 1988, intensive efforts have been taken to identify human ERα promoters. The regulation of ERα transcription is controlled by multiple promoters. So far, at least nine promoters have been discovered upstream of the translation start site of human ERα. A unified nomenclature for human ERα promoters was suggested by Gannon et al. [98]. The promoters of ERα contain multiple transcription factors-binding sites. The availability of these transcription factors may dictate the tissue-specific or context-dependent expression of ERα (Fig. 2a).

Previous studies demonstrated that estrogen receptor promoter B associated factor 1 (ERBF-1) is critical for the transcription activity of a distal promoter (promoter B) in ERα-positive breast cancer cells [99]. ERBF-1 is exclusively expressed in cells expressing ERα mRNA transcribed from promoter B and plays an important role in the expression of the ERα gene in breast cancer [100]. In addition, the transcription factor AP2 interacts with cis-regulatory elements via formation of dimers to regulate target gene expression. ERα expression is associated with AP2 activity in human breast and endometrial cancer [101]. The AP2 family proteins are recognized as key regulators in the development and progression of breast and endometrial cancer [102]. Both AP2α and AP2γ can trans-activate the human ERα promoter [100]. AP2γ recognizes a region in ERα promoter containing the sequence CCCTGCGGGG thereby inducing changes in the chromatin structure of ERα promoter and stimulating ERα transcription [103, 104].

The Forkhead box protein FOXO3a, which can be inactivated by Akt, is a positive regulator of ERα gene transcription [105]. However, FOXO3a interacts with ERα and ERβ proteins and inhibits ligand-dependent ER signaling and tumorigenesis [106]. FOXM1, another forkhead transcription factor, also regulates ERα transcription. FOXM1 activates the transcriptional activity of human ERα promoter primarily through two closely located forkhead response elements located at the proximal region of the ER promoter [107]. Reciprocally, FOXM1 protein and mRNA expression is regulated by estrogen, tamoxifen and fulvestrant in breast carcinoma. Depletion of ERα in MCF-7 cells down-regulates FOXM1 expression [108]. Moreover, FOXM1 and ERα can simultaneously bind to the same genomic sites and stimulate ERα transcriptional activity [109]. These finding suggest that ERα and FOXM1 may be two key components within a positive cross-regulatory loop.

The GATA proteins are a family of zinc finger DNA binding proteins that recognize the consensus motif T/A GATA A/G [110]. GATA-3 is highly expressed in T lymphoid cells and is a master regulator of immune cell function [111]. In the mammary gland, GATA-3 is expressed only by the epithelium and its expression increases during early pregnancy [111]. GATA-3 is an essential regulator of mammary gland morphogenesis and luminal cell differentiation [112]. The expression of GATA-3 is tightly correlated with ERα in human breast carcinoma [113]. GATA-3 binds to two *cis*-regulatory elements located within the *ERα* gene and stimulates ERα transcription [114].

While there are many transcription factors that positivily regulate ERα expression, little is known about the negative regulators of ERα transcription. The zinc finger repressor B-lymphocyte-induced maturation protein (BLIMP1) is able to bind to ERα promoter and inhibit ER transcription [115]. In addition, the transcription factor NF-*k*B can indirectly repress ERα transcription through inducing the expression of BLIMP1 and the Enhancer of Zeste Homolog2 (EZH2), which negatively regulates ERα transcription by inducing the di- and tri-methylation of histone 3 residue 27 [20, 115]. However, NF-*k*B enhances the recruitment of ERα to target DNA and increases the transcriptional activity of ERα [116]. Thus, the levels of ERα may be not always proportional to its activity.

There are several transcription factors that regulate ERβ transcription (Fig. 2b). The transcription factors c-jun and CREB can bind to ERβ promoter and promote ERβ transcription [117]. In addition, AP2α and AP2γ, two transcription factors that regulate ERα transcription, bind to ERβ promoter and stimulate ERβ transcription [118]. An evolutionally conserved E-box motif (CACGTG) has been identified in the ERβ promoter. Transcription factors containing the basic-helix-loop-helix (bHLH) protein structural motif typically bind to E-boxes or related variant sequences and enhance transcription of the downstream gene [119]. CLOCK and BMAL1 are members of the bHLH-PAS family of transcription factors that bind to E-box motifs and induce the transcription of target genes [120]. Both CLOCK and BMAL1 are positive set of components in an envolutionarily conserved feedback loop that controls the oscillation of circadian clock [120]. CLOCK-BMAL1 induces ERβ transcription via the E-box motif, whereas it does not regulate ERβ mRNA levels [121].

## Epigenetic regulation of ER expression

### ER promoter methylation

Gene expression may be silenced by methylation of a cytosine- and guanine-rich area, termed CpG island, in the promoter of the gene [122]. Methylation of CpG islands has been shown to inhibit transcription by preventing the binding of transcription factors to the promoter or by stabilizing structural changes in chromatin that prevent transcription [123]. The absence of ERα gene expression in ERα-negative breast cancer cells is associated with abnormal methylation in the CpG islands of multipe promoters of the ERα gene [124, 125]. Mechanistically, methylation of ERα promotet may prevent the recruitment of transcription factor such as AP2. In addition, ZEB1 can induce ERα promoter methylation, down-regulate ERα expression and promote anti-estrogen resistance in breast cancer [126]. Treatment of ERα negative human breast cancer cells with demethylating agents can induce partial demethylation of the ERα CpG islands and reactivate ERα gene expression [127]. Inhibition of DNMT1 by antisense oligonucleotides also caused ERα gene re-expression and the restoration of estrogen responsiveness in ERα negative breast cancer cells [128]. Demethylation of promoter C region in the ERα gene is in part responsible for the enhanced expression of ERα gene in long-term estrogen deprived MCF7 cells [129].

The expression of ERβ is also regulated by promoter methylation. Two promoters, promoter 0K and 0N, control the transcription of ER-β [130]. The lack of ERβ1, ERβ2 and ERβ4 transcription in some breast, ovarian and prostate cancer tissues and cell lines may be attributed to methylation of CpG sites in the promoter 0N [131]. In contrast, the 0K promoter is demethylated in malignant breast and ovarian cancer cells, as well as in normal breast and ovarian epithelial cells [132, 133]. Hence, ERβ promoter 0N methylation may be a target for manipulating ERβ expression.

### Histone modification and ER transcription

Gene transcription is also regulated by chromatin remodeling. The so-called histone code is important for dynamic regulation of chromatin assembly and gene transcription [122]. Chromatin structure is modulated by histone phosphorylation, acetylation, and methylation. Histone acetyltransferases (HAT) transfer an acetyl moiety to lysine residue on histones, leading to neutralization of the positive charge, reduced affinity of histone for DNA, and the transformation of a tight-coiled inactive chromatin structure into a loose, transcriptionally active one [134]. Histone acetylation also plays a role in ER expression. Treatment of ERα negative breast cancer cells with histone deacetylase (HDAC) inhibitors can restore ERα transcription [135]. Moreover, combination of DNA demethylating agents and HDAC inhibitors can induce ERα expression to more extent than treatment of ERα negative breast cancer cells with these agents alone [136]. Moreover, ERα expression is regulated by histone methylation in ERα enhancers. The H3K4 methyltransferase KMT2C up-regulates ERα through regulating H3K4me1 and H3K27ac at ERα enhancers [137]. Regulation of both enhancers and promoters may synergistically affect ERα transcription.

## Posttranscriptional regulation of ER expression

Transcription of the ERα gene gives rise to an mRNA that is 4.3 kb long and contains an extensive 3′ untranslated region (UTR) that is three-fold longer than its open reading frame [76]. The ERα 3′-UTR is known to contain several regulatory elements, including long tracts of AU-rich sequence and 13 copies of AUUUA [138]. AU-rich elements may direct mRNA destablization through mechanisms involving polyadenylase tail digestion and distributive deadenylation [139, 140]. Similar to AU rich elements in the 3′-UTR of other transcripts, the AU-rich elements in the 3′-UTR of ERα mRNA play critical roles in ERα mRNA destablization [137]. AUFp45 binds ERα mRNA and increase its stability by protecting it from RNAases [141]. In addition, the RNA-binding protein HuR plays a critical role in stablizing ERα mRNA [142]. It remains to know if there are other RNA-binding proteins that regulate the stability of ERα mRNA.

MicroRNAs (miRNAs) are small non-coding RNA that regulates gene expression at posttranscripton or translational level [143]. Both ERα and ERβ expression are regulated by miRNAs [144]. The expression of miRNA-206 is increased in ERα-negative tumors and it directly targets ERα by base pairing to the 3′-untranslated region of the ERα mRNA [145, 146]. miR-22, miR-130a, miR-17/92, miR-145 and miR-206 also directly target ERα mRNA and inhibits its expression [147, 148]. In addition, miR-27a indirectly regulates ERα expression by targeting ZBTB10, a repressor of specificity protein that regulates ERα expression [149]. Interestingly, some of the ERα-targeting miRNAs are also regulated by ERα. For example, ERα agonists downregulate miR-22, miR-206, miR-221, and miR-222 expression [150]. Moreover, miR-92 inhibits ERβ1 expression by direct targeting the 3′-untranslated region of the ERβ mRNA [151]. Certainly, there will be more ERβ-targeting miRNAs that may be uncovered in future studies. Furthermore, both ERα and ERβ regulate the expression of multiple miRNAs [152–156]. Because one miRNA is able to regulate many genes, ERα and ERβ may link multiple miRNAs to regulate the expression of a large pool of genes.

## Concluding remarks

In light of the critical roles of estrogen receptors signaling in diverse cellular processes and development, it is reasonable that the expression of ER and the activity of ER must be tightly regulated. Deregulation of ER is involved in tumorigenesis in multiple organ sites, including breast, ovary, endometrium and colon. ER expression can be regulated at multiple levels. Dynamic expression of ER is also a feature of human breast cancer. Even in ER-positive breast tumors, the expression of ER is not always permanent. Progression from an ER-positive phenotype to an ER-negative phenotype typically involves the constitutive activation of growth-promoting signals, thereby leading to a loss of estrogen dependence and resistance to anti-estrogens. This increased activation of growth factor receptors correlates with increased MAPK activity [156, 157]. Abrogation of MAPK activity can reverse the downregulation of ERα by growth factor signaling and restore its activity [158].

The detection of ERα expression in breast cancer is a routine practice in clinical setting. Given that there are multiple isoforms of ERα with different localization and functions, it may be necessary to discriminate which isoform is expressed in human breast cancer specimens. Moreover, the expression of ERβ isoforms should be detected. Detection of these isoforms may not only guide endocine therapy and/or other emerging therapeutics for breast cancer [159], but also help better judge the progmosis of cancer patients. Except for the levels of ER, the activity of ER may be more critical for the sensitivity to endocine therapy. The levels of estrogen responsive genes may reflect, at least in part, the activity of ER in human breast cancer. Currently, immunohistochemical analysis of PR positivity in human breast cancer is routine procedure in the clinic. Other estrogen responsive genes may also be detected to strenghten this facet.

Since ERα-negative breast tumors are less likely to be responsive to endocrine therapy, restoration of ERα expression could allow endocrine therapy to be effective in a subset of ERα-negative breast cancer. After reviewing the mechanisms underlying the regulation of ER expression, it is obvious that ER expression can be restored by multiple agents, including signal transduction inhibitors, monoclonal antibodies, DNA-demethylating agents or HDAC inhibitors. In addition, inhibition of Src can enhance ERα expression and anti-estrogen response by preventing ERα proteolysis [160]. Conversion of ERα-negative tumors to ERα-positive phenotype may allow an endocrine therapy that would prevent tumor progression. Whether or not these approaches can achieve clinical success remains to be determined.

## Abbreviations

AP: activating protein
E2: estradiol
ER: estrogen receptor
ERBF-1: estrogen receptor promoter B associated factor 1
ERE: estrogen response element
HDAC: histone deacetylase
IGF1: insulin-like growth factor 1
MAPK: mitogen-activated protein kinase
NF-*k*B: nuclear factor kappa-light-chain-enhancer of activated B cells
PR: progesterone receptor
